# Of the Use of Stimulants in Inflammatory Diseases of the Lungs in Children

**Published:** 1845-10

**Authors:** 

**Affiliations:** Berlin


*Of the Use of Stimulants in Inflammatory Diseases of the Lungs in Children.*—By Dr. Posner, of Berlin.—In inflammation of the lungs in of infants, the author admits a stasis of the blood in the capillary vessels, and from this circumstance he argues that we ought to remove the obstacles to the circulation of the blood. He first obtains this effect by the employment of sanguineous emissions, and the use of those medicines calculated to augment the fluidity of the blood. It is, however, necessary to allow to the circulation sufficient energy to carry out of the lungs the products of inflammation. If we carry the anti-phlogistic treatment too far, we incur the serious risk of inducing an asthenic inflammation. According to our author, a period may arrive when a stimulating treatment may be indicated. M. Posner, on this subject, observes, that the pneumonia of infants differ from those of adults. In the latter, we may bleed freely with advantage, and there is but little danger of inducing exhaustion; but in the case of children we must exercise more discretion; we must not push depletion too far, otherwise we sacrifice the life of the little patient. Under the influence of too abundant bleedings, they become pale and almost livid; their lips assume a bluish appearance; the face hippocratic; the pulse rapid; respiration hurried; the cough less frequent, but paroxysmal. Ausculta-tion reveals the râles characteristic of hepatization. If we in the anti-phlogistic system of treatment, we have symptoms toms of adynamia, and ataxia, convulsions, coma, and death; not from the effects of the disease, but from the treatment.

If, on the contrary, we decide promptly upon the employ-ment of stimulants, the patient may yet be saved. In such cases, our author extols wine, which the little patients now swallow with instinctive avidity. It should be given, at first, in small quantities, but persevered in regularly. It will soon impart a better expression to the face, diminish the fre-quency of pulse and the respiration, and induce a quiet and refreshing sleep. In a few hours, under this course of treat-ment, Mr. Posner has seen the râles of an hepatization changed into those of a simple catarrh. After the persistance of the anti-phlogistic treatment, and the use of calomel and stibiated tartar, for about two days, then our author thinks we may appeal to stimulants, and the cure will be certain and rapid. Before we resort to wine, we may try the polygala, and the ammoniacal preparation. Many cases are cited by Posner to confirm the views above developed.

*Remarks.*—The observations of the Prussian physician are, in our opinion, highly judicious, and deserve consideration. The advocates for depletion see nothing but inflammatory engorgement of the viscera, and believe the debility the result of a sthenic condition of the system; whereas, those who dread the lancet, see, on the contrary, nothing but asthenia, adynamia, and their fatal consequences. To both, we would say, bleed to fulfil certain indications, and stimulate when the symptoms seem to call for it. Exclusivism in medicine is a disgrace to the science, and should not be tolerated. Let practical medicine aspire to something more than a thing of fashion—to be patronized into notice to-day, and frowned down to-morrow.—*New Orleans Medical and Surgical Journal.*

